# Cervicovaginal microbiota: a promising direction for prevention and treatment in cervical cancer

**DOI:** 10.1186/s13027-024-00573-8

**Published:** 2024-04-19

**Authors:** Jie Shen, Hao Sun, Jing Chu, Xiaodi Gong, Xiaojun Liu

**Affiliations:** Department of Gynecology and Obstetrics, The Second Affiliated Hospital of Naval Medical University (Shanghai Changzheng Hospital), 200003 Shanghai, China

**Keywords:** Cervicovaginal microbiota, Cervical cancer, Human papillomavirus, Cancer treatment

## Abstract

Cervical cancer is a common malignancy in women, with high incidence rate and mortality. Persistent infection of high-risk human papillomavirus (HPV) is the most important risk factor for cervical cancer and precancerous lesions. Cervicovaginal microbiota (CVM) plays an essential role in the defense of HPV infections and prevention of subsequent lesions. Dominance of *Lactobacillus* is the key of CVM homeostasis, which can be regulated by host, exogenous and endogenous factors. Dysbiosis of CVM, including altered microbial, metabolic, and immune signatures, can contribute to persist HPV infection, leading to cervical cancer. However, there is no evidence of the causality between CVM and cervical cancer, and the underlying mechanism remains unexplored. Considering the close correlation between CVM dysbiosis and persistent HPV infection, this review will overview CVM, its role in cervical cancer development and related mechanisms, and the prospects for therapeutic applications.

## Introduction

Cervical cancer (CC) is the fourth most frequent cancer in women globally with estimated 604 000 new cases and 342 000 deaths in 2020 [1]. It is estimated to be approximately 553 000 new cases and 229 000 deaths worldwide in 2024 [2, 3]. Infection of high-risk human papilloma virus (HPV), mainly HPV-16 and HPV-18, is recognized as a significant carcinogenic factor of cervical cancer [4, 5]. Although 85 − 90% of high-risk HPV (hrHPV) infections can be spontaneously cleared within 6 months, a few HPV infections will still persist, leading to cervical intraepithelial neoplasia (CIN), also-called squamous intraepithelial lesion (SIL), and ultimately invasive cervical cancer [4, 6]. The fact suggests the presence of other factors involved in the development of cervical cancer.

Cervicovaginal microbiota (CVM), as a protective barrier for female reproductive system, plays an essential role in the defense against several primary and opportunistic pathogens including sexually transmitted infections (STIs) [7], especially HPVs [6]. A healthy CVM can form an acidic microenvironment in the vagina, conducive to maintaining the integrity of cervical epithelial and mucus barrier, protecting the host from pathogen invasions [6]. The dysbiosis of CVM, in company with changes in microbial compositions, metabolites and immune microenvironment, will damage the barrier and epithelial cells, and disrupt the immune responses against HPV infections [8], ultimately leading to the development of cervical cancer [9].

**Fig. 1:**
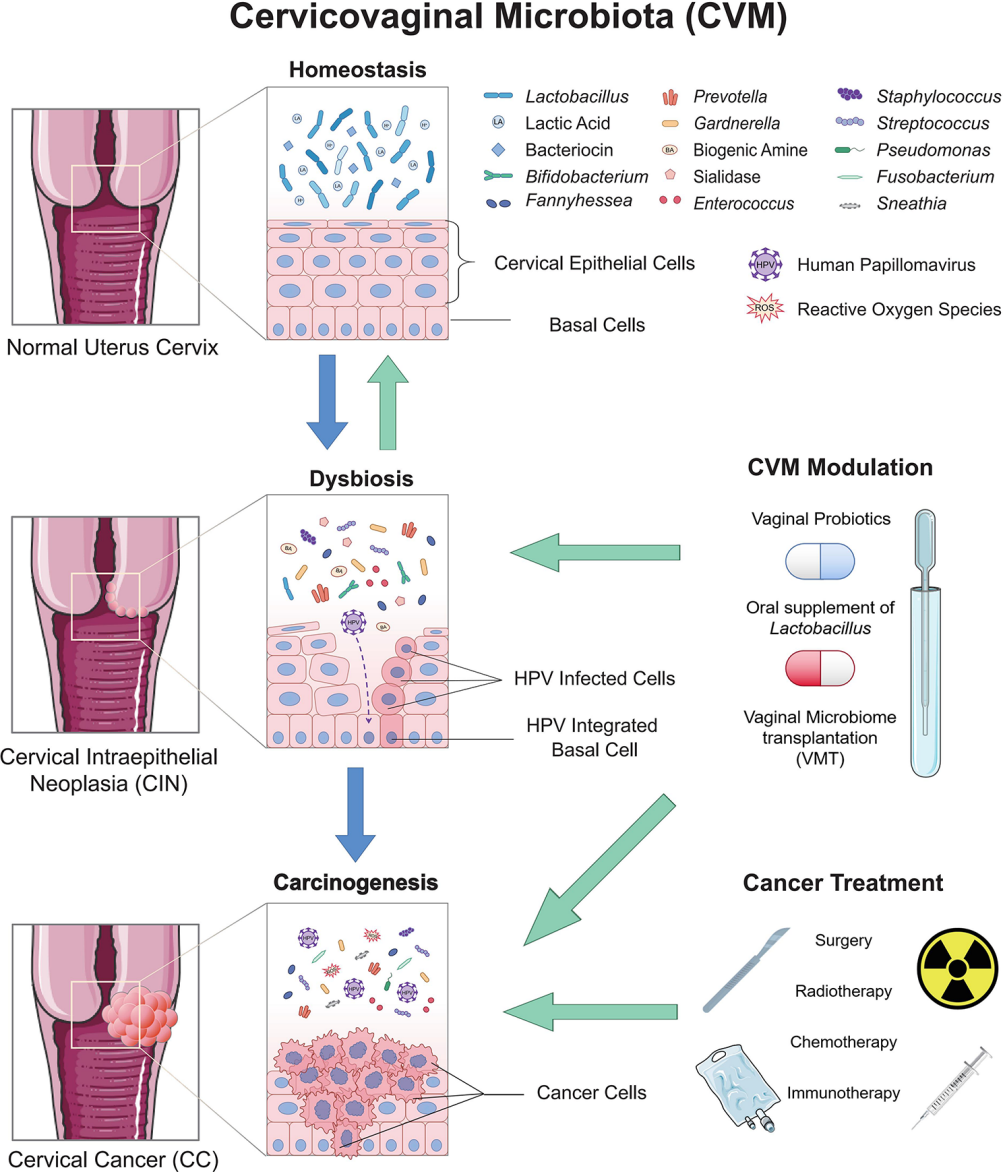
The role of cervicovaginal microbiota (CVM) in cervical cancer (CC). The homeostasis of CVM is dominated by *Lactobacillus*, forming an acidic microenvironment against pathogens invasion, such as HPV infection. Affected by host, exogenous, and endogenous factors, it turns to dysbiosis, a pro-inflammatory microenvironment with anaerobes dominance. Altered microbial, metabolic, and immune signatures lead to persist HPV infection and carcinogenesis. CVM differs in HPV/CIN/CC, so specific microbial species can be used as biomarkers. Modulation of CVM can enhance therapeutic efficacy, reduce adverse reactions and improve life quality. HPV: human papilloma virus. CIN: cervical intraepithelial neoplasia

The correlation among CVM dysbiosis, cervical HPV infection and cervical cancer progression has been widely proved [10, 11], while there is still no evidence for their causal links. Furthermore, little is known about the mechanisms how CVM participates in the disease progression. Thus, this review will overview the composition of CVM, its association with HPV infection and cervical cancer development, related mechanisms, and prospects for therapeutic applications (Fig. 1).

## Microbiota in the female reproductive tract (FRT)

### Construction and characteristics

Compared to the gastrointestinal microbiota, little is known about the role of reproductive tract microbiota in human diseases [12]. In contrast with other body sites, the reproductive tract harbors a microbiota with lower diversity, mainly dominated by *Lactobacillus* species [13]. Lactobacilli is vital for female reproductive health due to its probiotic activity in the microbiota [14]. Because of cervical mucus plugs, the female reproductive tract (FRT) can be divided into the lower FRT (vagina and cervix, with more microorganisms), and the upper FRT (uterus and oviduct, relatively sterile) [15]. Microbiota in lower FRT and upper FRT of the same female shows a continuity. With the rise in position, the overall biomass turns to decrease while microbial diversity gradually increases [16]. The cervicovaginal microbiota (CVM) is mainly dominated by *Lactobacillus* spp., while the abundance of *Lactobacillus* spp. in uterine microbiota (UM) is relatively lower [17].

### Composition of cervicovaginal microbiota (CVM)

**Fig. 2:**
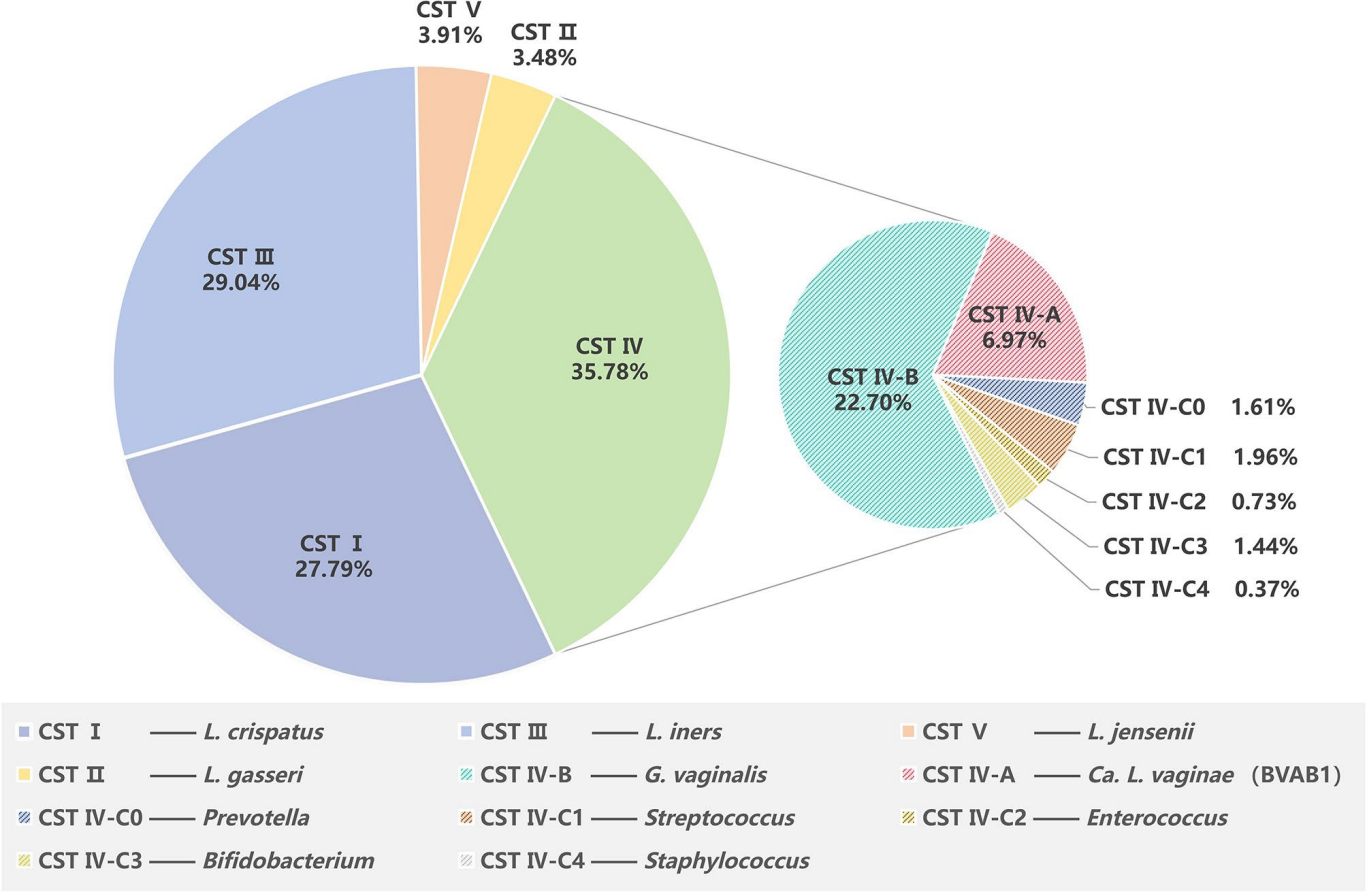
Representation of cervicovaginal bacterial community groups. CST: community state type

Generally, there is no significant difference between cervical microbiota and vaginal microbiota, jointly referred to as CVM [18]. Ravel et al. were the first to classify the CVM by microbial community structure [19] and had defined 5 community state types (CSTs). CST I, II, III, and V are dominated by a particular *Lactobacillus* species, respectively *L. crispatus*, *L. gasseri*, *L. iners* and *L. jensenii*. CST IV is a heterogeneous group typified by a combination of diverse facultative or strictly anaerobic bacteria and the depletion of *Lactobacillus*. CST IV has been subdivided into CST IV-A, IV-B, and IV-C [20]. CST IV-A has a high relative abundance of *Candidatus Lachnocurva vaginae* (formerly known as bacterial vaginosis-associated bacteria 1, BVAB1), while CST IV-B has a high relative abundance of *Gardnerella vaginalis*. Both IV-A and IV-B have moderate relative abundances of *Atopobium vaginae* (now reclassified as *Fannyhessea vaginae* [21]). CST IV-C has been divided into five sub-CSTs: CST IV-C0 is an even community with a moderate amount of *Prevotella*, CST IV-C1 is dominated by *Streptococcus*, CST IV-C2 is dominated by *Enterococcus*, CST IV-C3 is dominated by *Bifidobacterium* and CST IV-C4 is dominated by *Staphylococcus* (Fig. 2). Analysis of the relative abundance of bacteria revealed that the vagina and cervix showed high similarity in the microbial composition, suggesting ascending bacterial colonization from the vagina to the cervix, despite the cervical microbiota with a lower abundance of *Lactobacillus* and a higher abundance of *Prevotella* [22].

Sarah Lebeer et al. divided the CVM into four main modules of co-abundant cervicovaginal taxa based on compositional correlation network analyses, including *L. crispatus* module, *Gardnerella* module, *Prevotella* module, and *Bacteroides* module [23]. Some positive or negative correlations between the constituent taxa of these modules were observed, suggesting hidden interactions among the CVM components, though needed to be further experimentally validated.

### Uterine microbiota (UM)

Although the upper FRT used to be considered sterile, with the application of next-generation sequencing (NGS) technologies, recent studies have recognized the presence of UM and found a possible association between UM and female reproductive health [22]. Due to the low biomass and sample contamination during collection and processing, the identification of UM compositions varies significantly across studies. However, the lower relative abundance of *Lactobacillus* and higher microbial diversity of UM are observed in common [22, 24, 25], significantly different from the lower FRT microbiota. Generally, the community state of UM affects fertilization and pregnancy outcomes [24, 26]. However, most samples of UM are not from healthy individuals, so the exact composition of UM and its impact on the health of women and infants remains inconclusive [25].

## CVM Homeostasis and FRT health

CVM is a dynamic ecosystem in that its composition and relative abundance of bacterial species can fluctuate over short periods [27, 28]. Generally, a low microbial diversity with the dominance of *Lactobacillus* in CVM is considered as the homeostasis of CVM, beneficial to female reproductive health [29].

### What regulates the CVM

The composition of CVM varies in different women, due to some host-related factors, such as age, menstrual cycle, pregnancy status, genetic heterogeneity, racial and ethnic differences [19, 30–32]. Moreover, the homeostasis of CVM can be disrupted by some exogenous factors [23, 30, 33], such as using hormonal contraceptives and smoking, both related to cervical cancer development [34]. The microorganisms in CVM may interact with each other, regulate the CVM homeostasis by themselves [23].

The state of CVM is closely associated with the level of estrogen, as estrogen promotes glycogen accumulation in vaginal epithelial cells, providing the substrate for *Lactobacillus* to produce lactic acids [35]. The abundance of *Lactobacillus*, changes dynamically during the menstrual cycle with the fluctuation of estrogen levels [30, 36–38]. In CVM of adolescent or postmenopausal women, relatively lack of estrogen, the microbial species richness is decreased, but species diversity is increased significantly [39, 40]. The abundance of *Lactobacillus* spp. decreases, while the proportion of anaerobic bacteria increases [41]. In contrary, the CVMs in pregnant women are more stable than in non-pregnant women [26, 42], with lower microbial richness and diversity due to elevated levels of estrogen and progesterone [43, 44]. However, pregnant women are still more vulnerable because the increase of other bacteria being besides higher lactobacilli loads during pregnancy [14, 45].

However, the effect of hormonal contraceptives on CVM is debated. Some studies have suggested that synthetic estrogen in the compound oral contraceptives (COCs) is beneficial for *Lactobacillus* dominance [46–48], while others found no impact on CVM [49, 50]. The CVM become more diverse in the use of only progesterone (levonorgestrel intrauterine system, LNG-IUS) [51]. Moreover, COC use is associated with increased levels of inflammatory cytokines in the cervix [39, 52]. Such an inflammatory environment may relate to carcinogenesis [53, 54].

Behavioral factors can disrupt the CVM homeostasis. Smoking can induce a higher CVM diversity and the production of biogenic amines (BAs) in vagina [55], associated with pathogen invasions and vaginal malodor [56]. Menstrual hygiene, sexual behaviors, and childbirth are correlated with the presence of bacterial vaginosis-associated bacteria (BVABs) [23].

### Contribution of CVM to female reproductive health

#### Lactobacillus dominance

*Lactobacillus* is the most important component of CVM, playing an essential role in maintaining female reproductive health. Lactobacilli contribute to the reinforcement of the host immune system against several primary and opportunistic pathogens [57]. Lactobacilli produce lactic acid by fermenting glucose and maltose from vaginal epithelial cells, maintaining the vaginal pH at 3.8–4.5. Such an acidic microenvironment can inactivate pathogens and help regulate inflammatory responses [58], preventing pathogen invasions to the upper FRT [59]. Lactobacilli also secrete various metabolites that play antibacterial, antiviral, and immunomodulatory roles [60], such as bacteriocins, biosurfactants, and H_2_O_2_, inhibiting the proliferation of other microorganisms and the production of tumorigenic substances [61, 62]. The vaginal acidic environment is also beneficial to maintain the activity of bacteriocins and H_2_O_2_.

Although over 20 species of *Lactobacillus* have been detected in the vagina, CVM of most women is dominated by a single species of *Lactobacillus*, providing colonization resistance against pathogens, such as BVABs and HPVs [29]. The probiotic activity in CVM is caused not only by individual *Lactobacillus* species but also by its multi-microbial interaction as consortia [63]. Lactobacilli have a strong adhesion ability to the epithelial cells [64], which enables them to dominate the CVM and form a biological barrier, competing with pathogens for living space and nutrition [65]. Lactobacilli also can inhibit pathogenic adhesion and induce its displacement [66]. In addition, lactobacilli have a robust antimicrobial activity that can kill pathogens through direct contact [67].

The dominance of *Lactobacillus* can eliminate HPV infections, and even alleviate the progression of cervical lesions. Generally, CST I (*L. crispatus* dominance) and CST II (*L. gasseri* dominance) are the most frequent types in HPV negative women [68]. Moreover, CST II is associated with the most rapid clearance of acute HPV infection among HPV positive women [69]. Such homeostasis of CVM can be the protective factor for hrHPV clearance, with higher levels of soluble immunoglobulin A (sIgA), interleukin 2 (IL-2), and IL-1 in the cervicovaginal microenvironment [70].

Furthermore, lactobacilli and related metabolites adversely affect the growth and survival of cervical cancer cells [71]. The exopolysaccharides (EPSs), phosphorylated polysaccharides, and peptidoglycans secreted by the vaginal lactobacilli can inhibit the proliferation of cervical cancer cells and promote the process of apoptosis [72–74]. Studies have shown that lactobacilli and their supernatant had cytotoxic effects on cervical tumor cells but not on normal cervical epithelial cells [75], and were not affected by pH or lactic acid in the vaginal environment [76].

#### Bifidobacterium

Recent reports have identified the CVM dominated by *Bifidobacterium* in some healthy reproductive-aged women [77]. It is hypothesized that *Bifidobacterium* may provide a potential protective role similar to *Lactobacillus* in that it can also produce lactic acid and H_2_O_2_. To some extent, the proportion of *Bifidobacteria* and *Lactobacillus* may play a role in eliminating HPV infection [78]. Studies have indicated that some probiotics, such as *Bifidobacterium longum*, *Lactobacillus johnsonii*, *Lactobacillus plantarum*, *Lactobacillus fermentum*, and *Lactobacillus delbrueckii*, can inhibit various signaling pathways activated during HPV infection [79, 80], such as nuclear factor-κB (NF-κB) signaling pathway [81]. However, due to the scarcity of samples with *Bifidobacterium* dominance, more studies are needed to further clarify the clinical significance of *Bifidobacterium* in CVM [77].

#### Others

Generally, the abundances of other bacteria and fungi in the CVM, such as *Atopobium* and *Candida albicans*, are significantly lower than that of *Lactobacillus*, so the presence of these microorganisms usually causes no physical symptoms [82]. However, when the homeostasis of CVM is disrupted and the biological barrier formed by *Lactobacillus* no longer exists, these microorganisms, such as *G. vaginalis* and other anaerobes, will proliferate, develop biofilms, and cause recurrent female reproductive infections [83].

## CVM dysbiosis and HPV infections/carcinogenesis

The different CVM composition results in different susceptibility to HPV infections. Interaction of microorganisms and metabolites with host epithelial and immune cells can alter microenvironmental signatures, ultimately affecting defense against pathogen infections and disease progression [84]. In a healthy CVM, plenty of lactobacilli can maintain the low vaginal pH and produce bacteriocin, promoting an anti-inflammatory state in the vaginal epithelium, protecting its integrity and preventing the basal cells from HPV invasion [61].

When there is dysbiosis, lactobacilli are significantly reduced or absent, replaced by specialized or facultative anaerobic bacteria [85], weakening the vaginal defense. Enzymes secreted by the dysbiotic bacterial communities, such as sialidase, can disrupt mucus barrier [86], damage cervicovaginal epithelium [87], making the basal cells vulnerable to HPV infections [4, 88]. In addition, specific toxins from the bacteria can damage host DNA, leading to the integration of viral oncogenes into host genomes [13, 89, 90].

### Microbial composition and HPV persistence

The incidence and clearance rate of HPV infection varies in different CSTs [70, 91]. CST I is associated with lower HPV prevalence and higher detection rates of normal cells in cervical cytology [92]. In comparison, women with CST III and CST IV are 2–3 times more likely to be infected with hrHPV [93]. CST II (*L. gasseri* dominance) is associated with the fastest HPV remission rate, while CST IV-b (dominated by *Fannyhessea vaginae*), in contrast, shows the slowest remission rate [94]. The abundance of *Lactobacillus* is related to the clearance of hrHPV infection, while BVABs are linked with HPV persistence [95].

CST III and IV are believed to be less protective, associated with CVM dysbiosis, persistent HPV infection, and the development of cervical lesions [96].

#### CST III (*L. Iners* dominance)

*L. iners* is a transitional species that dominates the CVM after disturbance. *L. iners* is less able to inhibit colonization of pathogens. It can coexist with other bacteria in a wide range of pH and other metabolic stress-related situations [97, 98].

The limited protection provided by *L. iners* may be related to the fact that it can produce only L-lactic acid [99]. There are two isoforms of lactic acid. D-lactic acid has been reported to have a more significant inhibitory effect on exogenous bacteria than L-lactic acid [100]. *L. crispatus* (CST I) and *L. gasseri* (CST II) can produce both D- and L- lactic acid, *L. jensenii* (CST V) can produce only D-lactic acid, while *L. iners* lacks the gene that codes for D-lactate dehydrogenase (LDH) in its genome [101], resulting in a high L/D lactic acid ratio in CST III. The high L/D lactic acid ratio in vagina is related to the increase of extracellular matrix metalloproteinase inducer (EMMPRIN) and matrix metalloproteinase-8 (MMP-8), which are known to alter the tight junctions in the endocervical epithelium, making the female genital tract susceptible to infections [102, 103].

In addition, *L. iners* can produce inerolysin [104], a pore-forming cholesterol-dependent cytolysin (CDC), similar to the vaginolysin produced by *Gardnerella* [105]. It enables *L. iners* to obtain nutrients from host cells by creating aqueous pores within the cell membrane [104], which may disrupt the epithelial barrier. It can be hypothesized that the dominance of *L. iners* offers a favorable environment for pathogens like *Gardnerella* to survive and destabilize the CVM [102].

In cervical cancer, tumor-resident *L. iners*, as an obligate L-lactate-producing lactic acid bacterium, can alter tumor metabolism and lactate signaling pathways, causing therapeutic resistance and decreased survival in patients [106]. EMMPRIN and MMP-8, increaesd by high vaginal L/D lactic acid ratio, are also involved in cancer metastasis [103].

#### CST IV (non-*Lactobacillus* dominance)

CST IV, as a combination of diverse anaerobic bacteria, is often related to CVM dysbiosis [91].

Bacterial vaginosis (BV) is the most prevalent vaginal dysbiosis, characterized by a decrease in *Lactobacillus* and an increase in anaerobic bacteria, such as *G. vaginalis*, *Atopobium*, and *Prevotella* [65], which correspond to CST IV-A, IV-B, and IV-C0. BV leads to higher vaginal pH (above 4.5), vaginal malodor and irritation, pro-inflammatory vaginal environment and increased microbial diversity in CVM [107]. The inflammation activated by BV increases the levels of some cytokines that can be related to HPV infections [108–110]. As a result, BV is associated with adverse reproductive health outcomes and elevated risks for STIs (especially HPVs) [111].

BVABs (such as *G. vaginalis* and *Prevotella*) may contribute to HPV infection and persistence [91, 96]. Vaginolysin secreted by *G. vaginalis* can cause cellular lysis and tissue breakdown [105], promoting the integration of HPV DNA into keratinocytes. *Prevotella* species are also reported to link with HPV infection, as they increase the microbial diversity and disturb the CVM homeostasis by providing nutrients for other BVABs [112].

Aerobic vaginitis (AV) is another vaginal dysbiosis characterized by the loss of *Lactobacillus* and an increase in aerobes such as *Enterococcus*, *Escherichia coli*, *Staphylococcus*, and *Streptococcus* [113, 114], which correspond to CST IV-C1, IV-C2, and IV-C4. Similarly to BV, AV can also increase the risk of HPV infections.

### Altered metabolic signatures and carcinogenesis

CVM dysbiosis will alter the cervicovaginal metabolic profiles, conducive to HPV persistence [115]. A healthy metabolic microenvironment is characterized by high level of lactic acids, positively associated with the metabolism of lysolipids, phospholipids, glutathione, and glycogen, but negatively with the metabolism of biogenic amines (BAs), lysine, and histidine [116]. The *Lactobacillus* dominance is correlated with specific metabolites, such as anti-inflammatory nucleotides [115].

On the contrary, the metabolic microenvironment in women with BV is positively associated with BAs, lysine, and histidine metabolism, but negatively with lipid, glutathione, and glycogen metabolism. The levels of BAs (putrescine, cadaverine, and trimethylamine) and short-chain fatty acids (SCFAs) (especially acetate, butyrate, and formate) in BV are significantly high, while the levels of some amino acids (tyrosine and glutamate) in BV are relatively low [117]. In AV, the glycolytic metabolite GalNAc (N-acetylgalactosamine) and sucrose are downregulated, supporting the decrease of lactic acid [118]. The altered vaginal metabolic profiles can connect CVM dysbiosis to HPV infection and cervical carcinogenesis [115, 117].

#### Effects of biogenic amines (BAs)

BV is characterized by the loss of lactic acid and greater concentrations of mixed BAs (including polyamines putrescine, cadaverine, and trimethylamine) and SCFAs (including acetate, propionate, butyrate, and succinate), resulting in the higher vaginal pH and a pro-inflammatory vaginal environment [107].

Vaginal biogenic amines are the biomarker of BV, related to the vaginal malodor [119] and the reduction of *Lactobacillus* [120]. High production of BAs and nitrosamines leads to oxidative stress (OS) and nitrifying stress (NS). NS is associated with higher and greater pathogen resistance to the host defence systems, disturbing the immune responses [121]. OS is associated with numerous DNA lesions and protein modifications, contributing to carcinogenesis. Moreover, BAs may facilitate the formation of bacterial biofilms that entrap anaerobic bacteria, leading to their overgrowth and preventing the dominance of *Lactobacillus* [121].

#### Effects of short-chain fatty acids (SCFAs)

The effects of SCFAs are studied more in the gut than in FRT. SCFAs act as an energy source and immune modulator of the intestinal cell. Most studies show that SCFAs (especially butyrate) restore intestinal barrier function in inflammatory conditions by exhibiting anti-inflammatory effects in intestinal mucosa and inducing tight junction protein expression [122]. However, SCFAs appear to be pro-inflammatory in the FRT. BV organic acids (especially acetic and butyric acids) enhance the secretion of tumor necrosis factor α (TNF-α) after Toll-like receptor (TLR) 1/2/3 stimulation of cervicovaginal epithelial cells but inhibit the production of IL-6, RANTES (Regulated on Activation, Normal T cells Expressed and Secreted), and interferon-γ-induced protein 10 (IP-10) [123]. The difference may depend on the type and concentration of SCFAs, local pH and cell type, so the exact role of SCFAs in the FRT remains to be elucidated [124].

### Altered immune signatures and carcinogenesis

CVM dysbiosis can disturb the responses of host immune system by triggering inflammations, conducive to hrHPV infections [125]. The increased diversity of CVM leads to more production of cytokines and chemokines, amplifying the inflammation, and causing cell damage [89, 92–94]. The dysregulated immune response can create appropriate microenvironment for persistent HPV infection [60] and tumor development [89, 126].

Dysbiotic bacterial communities and their metabolites can stimulate local immune cells, leading to production of various inflammatory cytokines and reactive oxygen species (ROS) [127]. Acute inflammation may be protective for HPV clearance [8]. However, chronic inflammation and oxidative damage by ROS can exhibit genotoxic effects on epithelial cells [127, 128], consequently leading to cell apoptosis and tumourigenesis [89]. The dysbiotic microenvironment also contributes to cell proliferation, survival and migration, and angiogenesis, all of which are hallmarks of cancer [6, 89, 129]. However, it is still unknown whether CVM dysbiosis is involved in the immune escape of HPV [128]. Moreover, the long-term effects of CVM on host immune responses against cancer cells are barely studied.

#### Innate immune response

The CVM plays a significant role in shaping the immune response responsible for HPV clearance [8]. The bacterial or viral components are recognized by epithelial cells through TLRs, activating the innate immune response by releasing various pro-inflammatory cytokines [87, 128]. Macrophages and dendritic cells (DCs), as antigen presenting cells (APCs), are then activated and recruit immune effector cells, such as Natural Killer (NK) cells. APCs also stimulate antigen-specific T cells and B cells to activate the adaptive immune response.

CVM dysbiosis is associated with increased pro-inflammatory cytokines that can stimulate cell proliferation and promote the development of cervical cancer [108, 109]. Studies have shown that CST IV is related to the increase of IL-1α, IL-1β, granulocyte-macrophage colony-stimulating factor (GM-CSF) and IL-10 in cervix and vagina [130]. CST III is associated with increased IP-10 and monokine induced by interferon-γ (MIG) compared with CST I/II [130].

When it comes to specific cervicovaginal microbial species, *Lactobacillu*s usually plays a protective role in vaginal immunomodulation. *Lactobacillu*s has the capacity to improve antiviral defenses and modulate inflammation-mediated damage [108]. Lacobacilli can promote the epithelial cells to release surfactant proteins [57]. For example, *L. gasseri* LGV03 can significantly increase the production of interferon α (IFN-α) and IFN-β in HPV-positive cervical epithelial cells and reduce the expression of the pro-inflammatory cytokines like IL-6, IL-8, and IL-1β [71]. Lactic acid produced by *Lactobacillus* can act directly on the cervicovaginal epithelium, inducing the production of the anti-inflammatory cytokine, such as IL-1Ra, and reducing pro-inflammatory cytokine production [131]. In comparison, *G. vaginalis* or *Prevotella bivia* usually induce the increase of pro-inflammatory cytokines, like IL-6, TNF-α, IL-1α, and MMP-9 [108]. *Fannyhessea vaginae* and *Sneathia amnii* elicit more robust cytokine responses, including IL-6, IL-8, IP-10, monocyte chemoattractant protein-1 (MCP-1), macrophage inflammatory protein 3α (MIP-3α), RANTES, MMP-10, and MMP-1 [108]. Other BVABs, such as *Eggerthella*, only causes an increase in IL-1α; *Mobiluncus mulieri*s increases IL-1α, IL-6, IL-8, MCP-1, and TNF-α; while *Megasphaera micronuciformis* increases IL-1α, IL-1β, IL-1RA, TNF-α, and IL-6 [132].

These altered cytokines can serve as immune markers to predict BV status, HPV clearance, and CIN progression. It’s reported that high IL-1β/IP-10 ratio in BV is associated with lower rate of hrHPV clearance [110]. Elevated TNF-α/MIP-1β ratio in BV is prospectively associated with progression of persist HPV infections to CIN [110].

BVABs also stimulate the maturation and differentiation of APCs. *Megasphaera elsdenii* and *Prevotella timonensis* significantly promote the maturation of DCs, while the effects of *G. vaginalis* and *Lactobacillus* are not obvious [133]. *G. vaginalis* and its supernatants can induce THP-1 macrophages to differentiate into the M1 phenotype, which is involved in defence against bacterial infections, elevated ROS levels, and stimulation of the NF-κB/STAT1 (Signal Transducer and Activator of Transcription 1) pathway [134]. In contrast, vaginal *Lactobacillus* promotes M2 macrophages polarisation, which is involved in tissue repair and wound healing, helpful to restore the integrity of epithelial barrier [135].

The effects of bacteria on NK cells are rarely understood. NK cells are a critical component of the innate immune system, providing protection against a broad variety of viruses. Further studies are needed because the importance of NK cells in clearance of HPV-infected cells [136].

#### Adaptive immune response

The adaptive immune responses against HPV infection are most mediated by T cells. APCs activated by viral antigens can induce the effector CD8 + T cells targeting HPV-infected and neoplastic cells. CVM dysbiosis may induce a shift from anti-viral to anti-microbial immune response, resulting in HPV persistence [8, 137].

BVABs can act as pro-inflammatory factors, promoting the recruitment and differentiation of T cells [133]. *M. elsdenii* and *P. timonensis* significantly promote the differentiation of T cells into the pro-inflammatory Th1 type, with the increase release of IL-1β, IL-6, IL-8, IL-12p40, and TNF-α [133]. These cytokines can recruit Th1 and Th17 pro-inflammatory CD4 + T cells, effector memory CD8 + T cells, and leucocytes [138]. In contrast, *Lactobacillus* plays an anti-inflammatory role, promoting the differentiation of CD4 + T cells toward immunosuppressed Treg cells [139].

Reduction of lactobacilli and the less acidic environment may act as a pro-cancer factor, activating pathways related to cell proliferation and angiogenesis in the cervicovaginal epithelium [140]. In examination of immune mediators in local cervicovaginal microenvironment from women with or without cervical lesions, non-*Lactobacillus* dominance was associated with several pro-inflammatory (IL-36γ), chemotactic (IP10, MIP-1β and RANTES), haematopoietic (FLT 3 ligand) and adaptive immune response cytokines (IL-2, IL-4 and soluble CD40 ligand) [31]. In the cervicovaginal microenvironment of patients with cervical cancer, pro-inflammatory cytokines (IL-6, TNF-α), apoptosis-related proteins [soluble Fas receptor (sFas), sFas ligand, TRAIL (TNF-Related Apoptosis-Inducing Ligand)], growth and angiogenesis factors [hepatocyte growth factor (HGF), stem cell factor (SCF), vascular endothelial growth factor (VEGF)] and others [α-fetoprotein (AFP), osteopontin (OPN)] were elevated, positively correlated with vaginal pH, and negatively with the abundance of *Lactobacillus* [141].

## CVM and Cervical Cancer Development

### CVM and Oncogene expression

In cervical cancer, high-risk HPVs are essential for carcinogenesis and the maintenance of cancerous behavior. E6 and E7 are the main oncogenic protein of HPV, with the ability to bind and degrade tumor suppressor gene p53 and retinoblastoma protein (pRb) in infected host cells, causing cell proliferation out of control [85, 88].

The homeostasis of CVM can be disrupted by the expression of HPV oncogenes [142]. It has reported a two-way relationship between HPV infection and BV [143]. BV is a risk factor for HPV infection, and HPV infections are considered a cause for increased diversity, altered composition, and disordered function of CVM in turn [144]. Compared with HPV negative individuals, it is more likely to detect *Pseudomonas* [16], *Atopobium* [94, 96], *Fusobacterium* [92], and *Sneathia* [112] in CVM of HPV positive patients. Products of HPV E7 oncogene can significantly inhibit the expression of host defense peptides in the vagina (including HβD1, 2, 4, HD-5/6, SLPI, S100A7, and elafin) [143]. These peptides have antimicrobial activity against BVABs like *G. vaginalis.* Meanwhile, S100A7 and elafin expressed by the cervicovaginal squamous epithelial cells can be used as amino acid sources by lactobacilli for survival. Therefore, the survival of *Lactobacillus* species are considerably inhibited by HPVs, resulting in the CVM dysbiosis [143].

In turn, the expression of HPV oncogenes can also be regulated by the CVM in different stages of cervical lesions [85, 145]. The production of HPV oncoproteins is significantly upregulated during the progression of CIN. Along with the precancerous lesion development, the severity of CVM dysbiosis increased, including the increase of CVM diversity and richness, and the decrease of *Lactobacillus* [91, 146]. Various aerobic and anaerobic bacteria can be detected in the CVM with cervical lesions, such as *Gardnerella vaginalis, Prevotella bivia, Sneathia sanguinegens*, *Megasphaera micronuciformis*, and *Peptostreptococcus anaerobes* [147]. The expression of the HPV oncogenes is positively correlated with the presence of these microorganisms, but negatively with the presence of *Lactobacillus* [145]. Lactobacilli are reported to decrease the oncogene expression in cervical cancer cells, while *G. vaginalis* and *M. micronuciformis* can induce the production of viral oncoproteins [85].

### CVM as Biomarker for CIN and CC Detection

The microbial composition and abundance of specific species in CVM vary during cervical cancer development, which can be used to distinguish patients with HPV infection, CIN or CC. In a study with 5 groups [healthy, HPV positive (HPV+), low-grade SIL (LSIL), high-grade SIL (HSIL), and CC], the CC group showed the highest CVM diversity, significantly different from other groups [144]. The increase of the proportion of *Bacillus* and *Anaerococcus* and the decrease of the abundance of *G. vaginalis* may be related to the progression of CIN [144]. Another study showed that the abundance of *Gardnerella* was positively correlated with the CIN progression by inducing an increased CVM diversity over time, not directly causing HSIL [95]. An increased abundance of *Gardnerella* is not indicative of being pathogenic, but rather reflective of different bacterial relationships and host states [23]. Higher levels of *Sneathia sanguinegens*, *Anaerococcus tetradius*, and *Peptostreptococcus anaerobes* and lower levels of *L. jensenii* can be detected in CVM of women with HSIL than that with LSIL [147]. Comparing the CIN and CC groups, the presence of *Gardnerella* and *Streptococcus* differed significantly, with the former dominant in the CIN group and the latter dominant in the CC group [148]. The abundance of *Fusobacterium* and *Sneathia* is significantly higher in advanced CC than in the early stages, and *Fusobacterium necrophorum* is observed only in CC [92, 112]. The presence of *Fusobacterium* in CVM may lead to elevated cervical expression of anti-inflammatory IL-4 and transforming growth factor β1(TGFβ1), contributing to carcinogenesis [92].

Microbial species mentioned above can be potential biomarkers for HPV infection, CIN, and CC. However, the clinical significance of these biomarkers is limited because of the small sample sizes and no assessment of environmental factors, needed to be verified in further studies [70].

## CVM modulation and cancer treatment

The CVM homeostasis, with *Lactobacillus* dominance, is essential for female reproductive health. Besides its protective role in cervical cancer prevention, CVM can affect host responses to cancer treatment.

Treatment for cervical cancer, including surgery, radiotherapy, chemotherapy or a combination of chemoradiation, depends on the stage. Immunotherapy is a new option approved by the US Food and Drug Administration (FDA) [5]. For instance, the immune checkpoint inhibitor targeting programmed cell death-1 (PD-1) and programmed cell death ligand-1 (PD-L1) are used in advanced cervical cancer with progression during or after chemotherapy [5].

Therapies against cancer can disturb the state of CVM [149], inducing treatment-resistance [106]. Thus, the modulation of CVM, turning the dysbiosis back to homeostasis, will improve the therapeutic efficacy, providing a potential direction for cervical cancer treatment.

### Surgery

The state of CVM may be related to the recurrence of CIN or HPV infection after surgery. Effect of CIN excision on CVM is controversial. Some studies have observed a decreased CVM diversity and an increase in *Lactobacillus* after excision [150, 151]. However, another study reported no significant difference in CVM composition after treatment [152]. Further studies are needed to verify the association among the CVM state, CIN resection, and recurrence rates after treatment. The alternation of CVM before and after surgery in patients with cervical cancer, especially with or without the recovery of CVM homeostasis, can be essential to distinguish patients with high risk of recurrence.

### Radiotherapy

Radiation can disrupt the CVM communities [153, 154]. Women with gynecological cancer before and after radiotherapy showed an increase in *Mobiluncus*, *Atopobium*, and *Prevotella* and a decrease in *Gardnerella* and *Peptostreptococcus*, as well as *Lactobacillus* following treatment [153]. The microbial diversity of CVM in cancer patients also increased after radiotherapy [154]. Radiotherapy also causes epithelium damage, resulting in various adverse effects, such as vulvovaginal atrophy (VVA), vaginal stenosis, and pelvic pain [155]. A reduced amount of *Lactobacillus* is observed in patients with radiation-induced VVA [156]. In addition, the presence of certain microbial species, like *L. iners*, was reported to induce chemoradiation resistance, while *L. crispatus* did not [106]. Thus, modulation of CVM may relieve related adverse reactions and reduce radiation-resistance.

### Chemotherapy

Regarding chemotherapy, a study has found lower CVM diversity in CC patients can be associated with more significant responses to platinum drugs than non-responders [149], suggesting that modulation of CVM may enhance the chemotherapeutic efficacy, despite lack of exploration for mechanisms. In addition, studies on the gut microbiome reveal that microbiota can reduce the toxicity of chemotherapeutic agents and improve the efficacy of chemotherapy and immunotherapy [157].

### Immunotherapy

The role of CVM in immunotherapies is still unclear. However, studies show that the diversity and composition of the gut microbiome can affect anti-tumour immunity and the efficacy of PD-1 immunotherapy in various tumor types [158–160]. The mechanisms include translocation, immunomodulation, drug metabolism, and enzymatic degradation [157, 161]. Moreover, studies on gut microbiomes suggest that the interactions between bacteria and the host immune system may also play roles in patient responsiveness to immunotherapeutic agents [157–160, 162–164]. Structural and metabolic features of microbiota can regulate the immune response to cancer cells [165–167].

As HPVs are essential for carcinogenesis in cervical cancer, cancer vaccine targeting HPV is a kind of immunotherapy specific to cervical cancer. *Lactobacillus* can not only convey antigens as a vaccine carrier, but also enhance the immune response as a vaccine adjuvant. A *Lactobacillus*-based oral vaccine, expressing HPV E7 protein on the surface of *Lactobacillus casei* strain, is undergoing clinical trials, and has induced the regression of CIN [168].

### CVM Modulation

Microbiome-modulation can reduce the therapeutic toxicities, enhance the therapeutic efficacy and improve life quality for patients [157]. It also has essential health benefits in preventing the genital inflammations, STIs, and cancers.

Exogenous lactobacilli supplementation can somewhat reverse the dysregulation of CVM [169, 170]. Oral administration of a pertinent lactobacilli strain mixture can improve vaginal health in asymptomatic women with vaginal dysbiosis [171]. Furthermore, probiotics consisting of *Lactobacillus* spp. may increase the clearance of HPV and delay the progression of cervical cancer [172]. Long-term (6 months) use of *Lactobacillus rhamnosus* BMX 54 is twice as likely to resolve HPV-associated cytological abnormalities than short-term use (3 months) [173].

Moreover, the feasibility of vaginal microbiome transplantation (VMT) as the treatment for women with vaginal disorders has been proven [174]. However, the long-term effects remain unknown, and the potential risks remain elusive.

## Discussion

With the advance of sequencing technology, the composition of FRT microbial communities, specific bacterial species, and their contributions to health and disease have been preliminarily identified. However, the 16S rRNA sequencing technique still has some limitations, especially a lack of absolute bacterial quantification. Other molecular techniques for bacterial quantification, for example, quantitative real-time PCR or flow cytometry, could be used to determine absolute loads of specific bacteria associated with particular conditions [13, 110].

CVM is dynamic, and various factors can regulate its constructure and composition [30]. Some women with CVM abnormality complain no apparent symptoms. It is difficult to distinguish whether such CVM alteration is a normal fluctuation or an indication of female reproductive diseases. Longitudinal studies are required to evaluate the necessity of intervention.

Besides *Lactobacillus*, some studies have reported the dominance of other lactic acid bacteria in the CVM [77, 175]. However, whether these bacteria can provide the similar protective effects to *Lactobacillus*, such as the colonization resistance against pathogens, still remains to be explored.

The impacts of exogenous factors related to cervical cancer on CVM, like hormonal contraceptives and smoking, are controversial. It is hard to tell whether they increase the risk of CC by affecting CVM or promote the genesis of CC resulting in changes in CVM [56].

The development of cervical cancer is closely related to HPV infection. Although HPV infections, in general, are non-inflammatory, inflammatory reactions induced by CVM dysbiosis have been proven contributing to the development of CIN. Moreover, the long-term persistence of HPV is thought to result in generalized immunosuppression [121]. However, studies have reported that some BVABs, such as *G. vaginalis*, are involved in a shift from antimicrobial to antiviral responses, related to HPV clearance [8, 87]. In contrast, *Lactobacillus* plays an immunosuppressive role [139]. It is still unknown how CVM dysbiosis involves in the immune escape of HPV [128], and long-term effects of CVM on host immune responses against cancer cells are barely studied [121]. Notably, *Gardnerella* species are now divided into *Gardnerella vaginalis*, *Gardnerella leopoldii*, *Gardnerella piotii*, and *Gardnerella swidsinskii*, as these different species show distinct ecological or pathological properties [176]. The disputed pathogenic role of *G. vaginalis* may be the result of formerly considering different species as a single species.

Studies about mechanisms of CVM in cervical carcinogenesis are relatively rare. Integrated multi-omics approaches have been used to identify microbial and host signatures (bacterial communities and species, immune mediators and other proteins, and metabolites) in the cervicovaginal microenvironment. 3D cell cultures and mouse models are required in further research to determine the mechanism for the role of microbial communities or single specific microbial species with host-microbiota interactions in cervical carcinogenesis [108, 177].

Most studies about microbiota and carcinogenesis are focused on the gut microbiota. However, there are significant differences between the two body sites. There is an acidic environment in the vagina (pH < 4.5), while the pH of the intestinal environment is over 7.0. Besides, a healthy CVM is associated with low diversity, while in the intestine, high microbial diversity is considered a sign of health [178]. What’s more, the concentration and composition of microbial metabolites are different, leading to different effects on cells, which also differ depending on pH and cell types. In addition, considering that the CVM can be regulated by estrogen levels, and that circulating estrogen levels in the body can be influenced by the gut microbiota, there may be a connection between the gut and cervicovaginal microbiomes [156, 179]. The modulation of gut microbiome may contribute to the restoration of CVM homeostasis.

The interactions between CVM and cancer treatment have not been thoroughly studied. More studies are needed to verify the therapeutic efficacy affected by CVM and the recurrence rate of gynecological diseases after modulation of CVM.

## Conclusion

CVM is different from microbial communities in other body sites, with a low diversity and mainly dominated by *Lactobacillus*. However, its role in cervical cancer development and related mechanisms remains unclear. Homeostasis of CVM is crucial for maintaining female reproductive health, and such a dynamic ecosystem can be affected by host, exogenous, and endogenous factors. Dysbiosis of CVM, including changes in microbial, metabolic, and immune signatures, can form a pro-inflammatory microenvironment, weaken the resistance to pathogens (including HPVs), and contribute to carcinogenesis. Numerous studies have demonstrated the association of cervicovaginal dysbiosis with HPV infection, CIN, and CC. Besides, specific microbial species have been identified as biomarkers for HPV/CIN/CC. Several studies have explored the mechanisms of microbial interactions with HPV and cancer cells, but further research is still needed to confirm the influence of microbiota on disease in 3D cell cultures and mouse models. Cancer treatment can also affect CVM, and modulation of CVM can help enhance therapeutic efficacy, alleviate adverse reactions, and improve the life quality of patients. Understanding the role of CVM in cervical cancer development may provide new opportunities for cancer prevention, treatment, and improvement of female life quality and overall health.

## Data Availability

No datasets were generated or analysed during the current study.
